# Annotations

**Published:** 1899-03-11

**Authors:** 


### ANNOTATIONS.

### Municipal Amenities.

AN amusing instance of the silly spirit of antagonism which is sometimes allowed to grow up between public bodies whose first duty ought to be the promotion of the general good, is to be found in the archives of the Glossop Town Council as detailed in the *Glossop Chronicle* for February 24th. It appears that this town possesses an isolation hospital, which, however, is situated within the area of the Rural District Council. Now the nurse at this institution being attacked with typhoid fever in the course of her duties as nurse, and, therefore, as servant of the Town Council, was treated at their own hospital where the disease occurred, which to the ordinary mind would seem reasonable and proper. Not so, however, to some of the wiseacres of Glossop. Unfortunately there is a little feud between the Urban and the District Councils. The Urban Council has a quarry which is situated outside their own district, also some waterworks, and, sad to relate, like any other owners of property, they have to pay rates to the district in which their property lies. Hence many tears among a section of the Glossop Town Council, and hence also the delight of the said party when it occurred to one of them that, although the sick woman who was now suffering from typhoid fever had caught the disease in their service and in their own hospital, she evidently was resident all the time within the area of the District Council. Here was a fine chance! To make the Rural Council pay for the support of the Town Council's own nurse would, indeed, be spoiling the Egyptians! Fortunately, however, saner counsels prevailed. We only mention this ridiculous discussion to show the cantankerous spirit of antagonism which only too often prevails between neighbouring sanitary authorities, much to the detriment of the good work which they might do.

### The Rating of Hospitals.

IT certainly seems a hardship that out of the money voluntarily contributed by the public for the support of hospitals in London something between £8,000 and £9,000 should be paid away in rates every year. When the matter was brought before the London County Council last week, Dr. Collins very properly urged that even if hospitals were not assisted out of the rates they should at least be left unrated. It is, perhaps, impossible to prove that the hospitals in doing their work of mercy save the rates, but nevertheless we may reasonably feel confident that such is the case; indeed, we must have a very poor opinion of the usefulness of these institutions if we hesitate to admit that by restoring sick people to health, by giving them back their working power, by shortening the time during which those who are dependent upon the bread-winner are thrown during his illness upon the hands of others, and by nipping in the bud that downward drift to pauperism which is so frequently the outcome of accident and sickness among the poor, the hospitals not only prevent people from becoming a burden upon the rates, but add to the length of the useful rate-paying life of many a good citizen. People are, perhaps, too apt to regard hospitals as places where extraordinary operations are performed and wonderful cures effected on out-of-the-way cases, although in a sense this view of the matter is right enough. But it is in their ordinary daily humdrum work that the hospitals save the rates. On the wealth of a town depend the rates, and this wealth in turn depends upon the steady labour of each member of the community. Nothing interferes with steady labour more than accident and sickness, and it is by their ever open door, by their immediate readiness to deal with the beginnings of illness, and by their thorough equipment for quickly restoring to the injured workman his capacity for work, that the hospitals add so greatly as they do to the wealth of the community. So certain is this and so obvious is their public utility that if, by evil chance, the hospitals should ever be deprived of the voluntary support which they fortunately now enjoy, they would have to be supported at the expense of those very rates which at the present time so ruthlessly levy toll upon them. It may be impossible without an Act of Parliament to put a stop to the rating of hospitals. But there are many ways of killing a dog besides drowning it, and no doubt there are many ways of putting an end to the oppressiveness of rates without calling in the powers of the Legislature to abolish them altogether. By all means let us have an Act exempting hospitals from rates—if such be possible; but, in the meantime, if hospitals must be rated let us deal gently with them, let us turn an unobserving eye to the minutiæ of assessment, and let us wink at short weight rather than force ourselves to demand our full pound of flesh from institutions which do so much to promote the public weal.

### Hospitals Without House Surgeons.

THE position taken up by the General Medica Council in regard to the employment of unqualified assistants is a matter that should receive the careful consideration of all medical men who are connected with cottage hospitals where house surgeons are not employed, for it is quite clear that the staff of a hospital have no right to do collectively what in an individual would be regarded as "infamous conduct in a professional respect." It behoves them then to take care that the arrangements for obtaining medical assistance in emergencies, and the limitations placed upon the work undertaken by the institution, are such that no unqualified person shall be called upon to act as an assistant, and in so doing to exercise professional discretion or skill. If a medical man opens a branch at some distance from his home and leaves it in charge of an unqualified person, who attends to slight cases, he undoubtedly does what the Council has decided is infamous in a professional respect. The fact that he goes there every day by no means relieves him from this charge so long as the unqualified assistant person is left in the position of having to exercise professional discretion and skill; and if the medical staff of a hospital depute a matron or other unqualified person to decide whether cases are urgent or not, and to deal with accidents on her own responsibility alone, they certainly lay themselves open to the same accusation. The only difference is that what is done in the one case is done by an individual, while in the other it is done by the staff collectively and acting through a committee. But we fail to see why medical men, acting as the staff of a hospital should be allowed to do what they would not be permitted for a moment to do in their individual capacity.

